# MoS_2_-clad microfibre laser delivering conventional, dispersion-managed and dissipative solitons

**DOI:** 10.1038/srep30524

**Published:** 2016-07-26

**Authors:** Yudong Cui, Feifei Lu, Xueming Liu

**Affiliations:** 1 State Key Laboratory of Transient Optics and Photonics, Xi’an Institute of Optics and Precision Mechanics, Chinese Academy of Sciences, Xi’an 710119, China; 2 State Key Laboratory of Modern Optical Instrumentation, Department of Optical Engineering, Zhejiang University, Hangzhou 310027, China

## Abstract

Molybdenum disulfide (MoS_2_), whose monolayer possesses a direct band gap, displays promising applications in optoelectronics, photonics, and lasers. Recent researches have demonstrated that MoS_2_ has not only a significant broadband saturable absorption performance, but also a higher optical nonlinear response than graphene. However, MoS_2_ shows much lower optical damage threshold owing to the poorer thermal conductivity and mechanical property. Here, we exploit a MoS_2_-clad microfibre (MCM) as the saturable absorber (SA) for the generation of ultrashort pulses under different dispersion conditions. The improved evanescent field interaction scheme can overcome the laser-induced thermal damage, as well as take full advantage of the strong nonlinear effect of MoS_2_. With the MCM SA, conventional, dispersion-managed, and dissipative solitons are generated around 1600 nm in Er-doped fibre lasers with anomalous, near-zero, and normal cavity dispersions, respectively. Our work paves the way for applications of 2D layered materials in photonics, especially in laser sources.

Ultrafast mode-locked fibre lasers have widespread applications as diverse as ophthalmology, micromachining, medical imaging and precision metrology^1^,^2^,^3^, because of their compactness, high stability, low cost, and easy turnkey operation^4^,^5^,^6^. The pulse evolution dynamics in the mode-locked fibre laser depends on the cavity dispersion map resulting in different output pulse properties^7^,^8^,^9^,^10^,^11^,^12^,^13^. Under the anomalous dispersion, the conventional solitons (CSs) with typical Kelly sidebands are generated from the balance between fibre dispersion and nonlinear Kerr effect^10^,^12^. When the positive and negative dispersion components form a near-zero dispersion laser cavity together, dispersion-managed solitons (DMSs) can be achieved^7^,^9^. DMSs experience the periodic stretch and compression, and have a smooth Gaussian-shape spectral profile^9^. As a result of the mutual interaction among the normal cavity dispersion, fibre nonlinearity, gain, loss and the spectral filtering, dissipative solitons (DSs) can be achieved in fibre lasers with net normal or all normal cavity dispersion^7^,^8^,^14^. In comparison with the CS fibre lasers where the pulse energy is limited at tens of picojoules, the DS fibre laser can generate strongly chirped optical pulses with much larger energy^7^,^14^.

In general, ultrashort pulses are produced using a passive mode-locking technique^4^,^6^,^15^. Numerous SAs have been proposed to implement the mode-locked fibre laser, such as nonlinear polarization rotation (NPR)^16^,^17^,^18^, nonlinear optical loop mirror (NOLM)^19^,^20^, semiconductor saturable absorber mirror (SESAM)^4^,^21^, single-walled carbon nanotubes^22^,^23^,^24^, graphene^25^,^26^,^27^,^28^, and other 2D layered materials^29^,^30^,^31^,^32^,^33^. Currently, SESAM is the dominant scheme, which can provide saturable absorption with various characteristics^4^,^34^. However, SESAM is very expensive and suffers from the low damage threshold^26^. NPR and NOLM have the all-fiber structure which can sustain the high laser power, while the mode-locking threshold is high^18^,^19^,^35^. Moreover, they are highly sensitive to the environment disturbance^2^,^19^. Graphene has been demonstrated as the saturable absorber at 1^28^,^36^, 1.55^27^,^37^, 2 μm^38^,^39^. Mode locking operations at different dispersion regimes have been reported with graphene-based SA^27^,^40^. In recent years, molybdenum disulfide (MoS_2_) whose monolayer possesses a direct band gap, displays promising applications in electronics, optoelectronics and photonics^41^,^42^,^43^. By contrast with graphene, MoS_2_ is demonstrated a higher saturable absorption response^44^, and has been utilized as an effective mode locker in a broadband wavelength^31^,^45^,^46^,^47^. However, MoS_2_ shows much lower optical damage threshold than graphene owing to their poorer thermal conductivity and mechanical property^15^,^45^,^48^. To circumvent the optical damage of MoS_2_, an evanescent field interaction scheme of the propagating light with MoS_2_ on a D-shaped fibre has been used to start mode-locking operation^49^. Recently, an all-surface technique that a monolayer graphene was wrapped around a microfibre can guarantee the maximum efficiency of the graphene nonlinearity, and it is employed as a SA in a mode-locked fibre laser for the generation of CSs^50^,^51^. In addition, DMSs have not been reported with MoS_2_-based saturable absorber until now.

In this article, monolayer MoS_2_ wrapped around a microfibre is used as a SA shown in Fig. 1(a), not only overcoming the laser-induced thermal damage, but also effectively exploiting the strong nonlinear effect of MoS_2_. With the MoS_2_-clad microfibre (MCM) SA, CSs, DMSs and DSs are generated around 1600 nm in Er-doped fibre lasers. With dispersion management technique employed, the fibre laser cavity dispersion can be normal, near-zero and anomolous dispersion when single mode fibres (SMFs) with different lengths are introduced into cavity. Under different cavity dispersion, pulses with distinct temporal and spectral profile are obtained, implying the different evolution dynamics. The experimental results confirm the effectiveness and practicability of the improved evanescent field interaction scheme, and potentially give some new insights into graphene-like materials related applications

## Results

### MoS_2_-clad microfibre SA

The microfibre is manufactured using the fused biconical taper process with a minimum diameter of ~7 μm. The monolayer MoS_2_ (from SixCarbon technol.) is grown on SiO_2_/Si substrate. Compared with the previous works^51^, the fabrication procedure is improved to reduce the residues attached to microfibre during fabrication. Firstly, polymethylmethacrylate (PMMA) is spin-coated uniformly onto MoS_2_/SiO_2_/Si and dried. The resulted PMMA/MoS_2_/SiO_2_/Si is cut into strip samples with the width of ~0.3 mm and the length of ~4 mm. Then MoS_2_ strips are separated from the substrate in the potassium hydroxide (KOH) solution, and the etchant and residues are removed in the deionized water. Finally, MoS_2_ strips are transferred and wrapped tightly onto the microfibre with assistant of alcohol. The detailed fabrication procedure of the MoS_2_-clad microfibre is provided in the Methods section.

**Figure 1:**
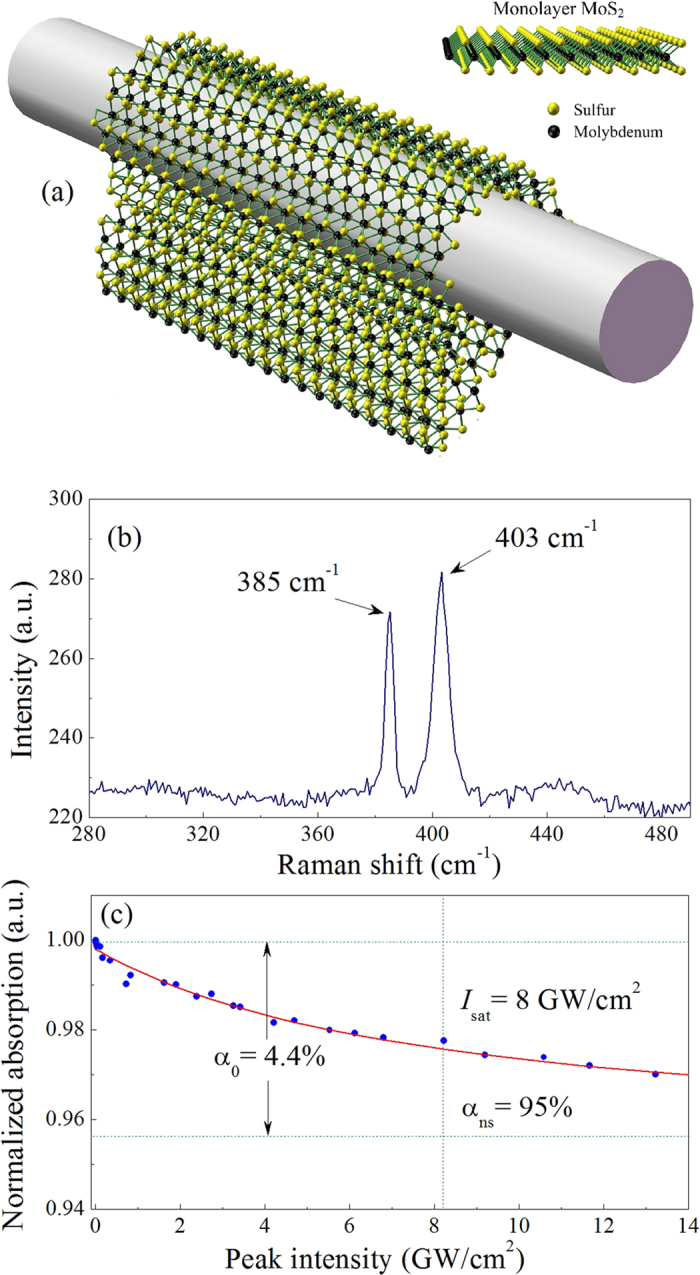
(**a**) Schematic diagram of the MoS_2_-clad microfibre saturable absorber (MCM SA). (**b**) Raman spectrum of MoS_2_. (**c**) Nonlinear absorption characterization of the MCM SA. The solid curve is a fit to the experimental data.

The samples are characterized by Raman spectrometry as shown in Fig. 1(b). Strong shifted peaks are observed at ~385 cm^−1^ and ~403 cm^−1^ corresponding to an in-plane E_2g_ vibrational mode and an out-of-plane A_1g_ vibrational mode of the layered MoS_2_ samples, respectively^31^,^35^. The spacing between the two peaks is ~18 cm^−1^, which reflects the characteristic of monolayer MoS_2_^15^,^41^. Figure 1(c) shows the normalized nonlinear absorption of MCM SA. The experiment is implemented with a homemade ultrafast fibre laser centred around 1570 nm with a pulse duration of ~240 fs. As shown in Fig. 1(c), the experimental data are fitted on the basis of a simplified two-level saturable absorption model^2^. Figure 1(c) illustrates that the linear limits of the saturable absorption (α_0_), the nonsaturable absorption (α_ns_), and the saturation intensity (*I*_sat_) are approximately 4.4%, 95%, and 8 GW/cm^2^. respectively. It should be noted that the saturable intensity is relatively high in our work, which could be attributed to the device where light interacts with MoS_2_ via evanescent field of microfiber. As discussed in ref. 51, the power density at the surface of microfiber is much smaller than that in the microfiber. Moreover, the power density at the surface of microfiber decreases exponentially with the microfiber diameter^50^,^51^. Here, a microfiber with the diameter of ~7 μm is used. The peak power density in the microfiber is employed to demonstrate the absorption of MCM SA. Yan *et al*. implemented the passively mode-locked fiber laser based on WS_2_ which was deposited onto a microfiber^52^. The intrinsic features of WS_2_ were shown with the saturable intensity of 25 MW/cm^2^ 52.

### Ultrafast fibre lasers based on MCM SA

**Figure 2:**
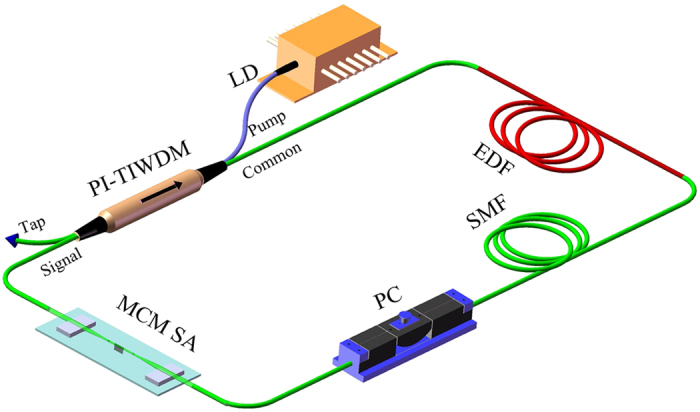
Laser setup. EDF, erbium-doped fibre; PI-TIWDM, polarization-independent tap-isolator-wavelength-division multiplexer; PC, polarization controller; LD, laser diode; SMF, single-mode fibre; MCM SA, MoS_2_-clad microfibre saturable absorber.

A schematic of the fibre laser mode-locked by MoS_2_ is shown in Fig. 2. The fibre laser system is composed of a 50-m-long erbium-doped fibre (EDF), a MCM SA, a polarization controller (PC), a section of standard SMF, and a compact integrated fibre device combining wavelength-division multiplexer, optical coupler and isolator. The usage of polarization-independent tap-isolator-wavelength-division multiplexer (PI-TIWDM) dramatically simplifies the fibre laser structure. The EDF and SMF have dispersion parameters of approximately −25 and 17 ps/(nm·km), respectively. The used EDF can provide large normal dispersion, and the cavity dispersion can be controlled by splicing SMFs of different lengths.

**Figure 3:**
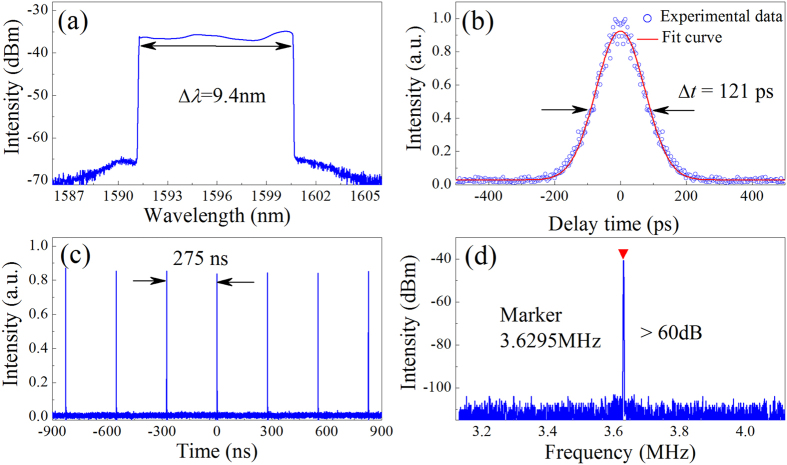
Typical laser characteristics of DSs. (**a**) Optical spectrum with a spectral resolution of 0.02 nm. The spectral width Δλ is approximately 9.4 nm. (**b**) Autocorrelation trace of the experimental data (circles) and Gaussian-shaped fit (solid curve). (**c**) Oscilloscope trace with a pulse separation of ~275 ns, corresponding to the cavity length of ~56 m. (**d**) Fundamental RF spectrum with a resolution of 1 kHz and a span of 1 MHz.

When only the fibre pigtails are used, the cavity length is ~56 m containing 50-m EDF and 6-m SMF. At this time, the total cavity dispersion is ~1.5 ps^2^. At the pump power of ~50 mW, continuous wave is established. With the appropriate pump power of ~130 mW and state of PC, stable mode-locking operation can be achieved. In this case, the average output power is ~1.8 mW. Once the pulse operation is obtained, no further PC adjustment is needed. A typical DS optical spectrum with a quasi-rectangular profile can be observed on optical spectrum analyser (OSA), as shown in Fig. 3(a). The centre wavelength is ~1596 nm with the spectral bandwidth of 9.4 nm. The second harmonic generation autocorrelation trace is illustrated in Fig. 3(b). The full width at half maximum (FWHM) is 171 ps, and the pulse duration is given as 121 ps if a Gaussian fit is used. The time-bandwidth product (TBP) is calculated as 134, which indicates that the pulse is highly chirped which is attributed to the large normal dispersion from the intracavity EDF. The pulse train depicted in Fig. 3(c) shows that the separation between adjacent pulses is ~275 ns, which is equal to the cavity round-trip time. The radio-frequency (RF) spectrum with a span of 1 MHz is shown in Fig. 3(d). The fundamental repetition rate of DS is ~3.63 MHz with the peak-to-background ratio more than 60 dB, implying a low-amplitude fluctuations and good mode-locking stability.

**Figure 4:**
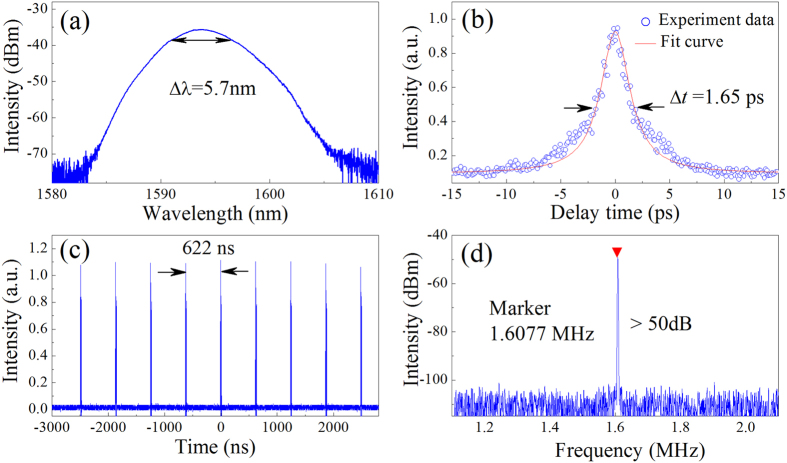
Typical laser characteristics of DMSs. (**a**) Optical spectrum. The FWHM spectral width Δλ is approximately 5.7 nm. (**b**) Autocorrelation traces of the experimental data (circles) and fit curve. (**c**) Oscilloscope trace with a separation of ~622 ns, corresponding to the cavity length of ~128 m. (**d**) Fundamental RF spectrum with a resolution of 1 kHz and a span of 1 MHz.

A section of standard SMF is spliced into laser cavity to adjust the net dispersion value. As lengthening the SMF, the cavity dispersion is gradually changed from normal to near-zero then to anomalous. In the experiment, when ~70-m SMF is used, pulses with smooth Gaussian-shape spectrum can always be obtained by appropriately adjusting PC state and pump power. DMS can self-start at ~90 mW and the output power is ~0.35 mW. Figure 4(a) shows the output spectrum centred at 1594 nm, which has a bandwidth of 5.7 nm. Figure 4(b) illustrates the corresponding autocorrelation trace whose profile indicates that the measured DMSs have been compressed. The small satellites on the autocorrelation trace results from the nonlinear chirp in the pulse^53^, as the pulses actually circulate along the long EDF and SMF. The pulse duration and the TBP are estimated as 1.65 ps and ~1.1, respectively. The oscilloscope trace in Fig. 4(c) shows that the pulse-to-pulse separation is ~622 ns corresponding to the cavity length of ~128 m. As depicted in Fig. 4(d), the fundamental repetition rate of DMSs is given as 1.6077 MHz. The peak-to-background ratio is more than 50 dB. Our works may demonstrate the first example of MoS_2_-mode-locked DMSs.

**Figure 5:**
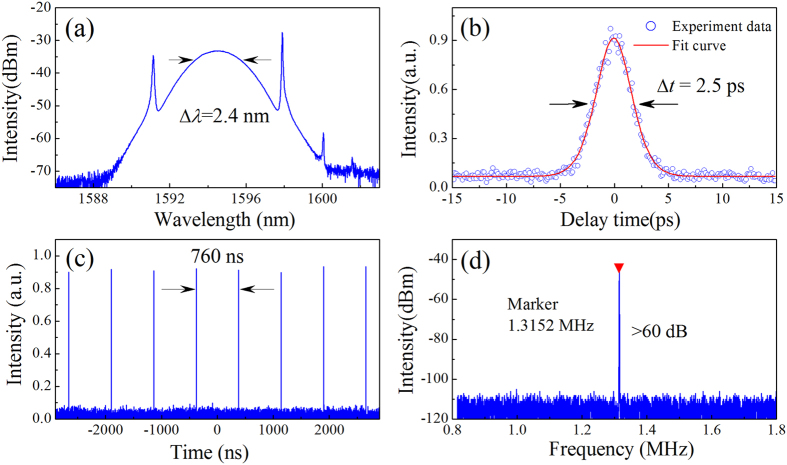
Typical laser characteristics of CSs. (**a**) Optical spectrum. The spectral width Δλ is ~2.4 nm. (**b**) Autocorrelation traces of the experimental data (circles) and sech^2^-shaped fit (solid curve). (**c**) Oscilloscope trace with a separation of 760 ns, corresponding to the cavity length of 156 m. (**d**) Fundamental RF spectrum with a resolution of 1 kHz and a span of 1 MHz.

When the intracavity SMF is further lengthened, spectral sidebands appear on the both sides of optical spectrum, which characterized the CS operation^12^. The sidebands stem from the constructive interference between the soliton and dispersive waves, when soliton suffers from periodic perturbations^7^,^10^. A self-started mode-locking operation is measured with ~100-m SMF. When the pump power is decreased to ~55 mW, single pulse operation can be obtained with the output power of ~20 μW. Figure 5(a) shows the typical laser spectrum with a centre wavelength of ~1595 nm and 3-dB bandwidth of 2.4 nm. Figure 5(b) illustrates the autocorrelation traces. Assuming a sech^2 ^profile, the deconvolution yields the pulse duration of ~2.5 ps. TBP is calculated as 0.7, meaning that the pulses are slightly chirped. Figure 5(d) shows that the repetition rate of the fundamental cavity frequency is 1.315 MHz, corresponding to 760 ns of round-trip time in Fig. 5(c). The RF spectrum gives a signal-to-background ratio of >60 dB, indicating low-amplitude fluctuations and good mode-locking stability^54^. The CS results also confirm the DMS operation where the spectral sidebands is absent with shorter SMF, *i.e.* smaller anomalous dispersion.

## Discussion

In recent studies, MoS_2_ shows the layer-dependent optoelectronic properties^41^,^43^. The bulk MoS_2_ has an indirect bandgap of 1.2 eV, and the monolayer MoS_2_ is a direct gap semiconductor with a bandgap of 1.8 eV^41^. However, the photon energy around 1600 nm (~0.77 eV) is below the bandgap. In fact, the edges, defects and boundaries of the mono- or few-layer MoS_2_ would induce a modification of absorption wavelength^15^. According to the explanation based on atomic defects, S-atomic defects can reduce the bandgap to ~0.08 eV, supporting the broadband operation wavelength of MoS_2_^15^,^31^. We demonstrate an efficiency scheme of incorporating MoS_2_ into fiber laser, which exhibit many merits. By comparison with SESAM, MCM SA is much cost-effectiveness and the evanescent field interaction scheme gives a high damage threshold. In the experiment, MCM SA can initiate mode locking at a lower pump power than NPR and NOLM. Self-started soliton operation can be achieved, which is independent of polarization state. However, the state of PC can influence the characteristics of pulses in the experiment, because the imperfects during fabrication procedure would induce the polarization-dependent loss. But it is much smaller than that of the common polarizer ( >20 dB). The insertion loss of MCM is less than 1.5 dB, and the loss variation with the polarization is within 0.5 dB. They hardly play such a role in the pulse generation dynamics. The self-started pulse operation and low mode-locking threshold indicate that the MCM SA dominates the mode-locking operation in this laser.

**Figure 6:**
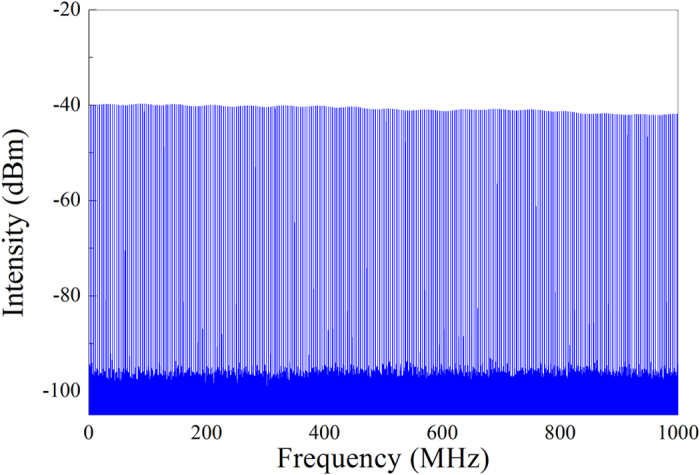
Wideband RF spectrum up to 1 GHz by taking DS for example.

It should be noted that three types of solitons can be obtained in a wide range of cavity parameters, for instance, CS is achieved with intracavity SMF of even several hundred meters. Under different dispersion map, fibre lasers emit pulses with distinct properties, such as spectral profile, bandwidth, pulse width and repetition rate, which imply that the intracavity pulse evolution is of difference though the same MCM SA is used. These results confirm the capability of the MCM-based mode locker of initiating pulse operation. It should be admitted that the laser cavity length is relatively long comparing with other general fiber lasers^2^,^12^,^24^. The influence of long cavity length is discussed as follows. Firstly, the long cavity length means the low repetition rate and the long round-trip time. With low repetition rate, the high single pulse energy can be obtained^16^,^17^. Secondly, the long intra-cavity EDF and SMF could introduce large dispersion and nonlinearity, which can induce much richer phenomenon and generate pulses with various features. Rectangular-shape pulses, soliton rains and dissipative soliton resonant have been reported in fiber laser with a long cavity length^8^,^36^,^55^. Thirdly, fiber laser with long cavity length is generally sensitive to the environmental fluctuation, such as the temperatures, strains and vibrations. To guarantee the stability of fiber laser, there needs better operation conditions. To further verify the stability of mode-locking operation, a wideband RF spectrum up to 1 GHz corresponding to DS is shown in Fig. 6, which is similar to these of CS and DMS. No spectrum modulation is observed, thereby indicating no Q-switching instabilities^12^,^54^.

In addition, CSs, DMSs and DSs are generated around 1600 nm which is located at L band. The use of a long EDF (~50 m) is capable of operating at longer wavelengths which have been used to achieve amplification at L band^56^. Zhao *et al*. have reported mode-locking operations at L band in an erbium-doped fiber ring laser based on NPR technique^57^. Recently, it has been made the L band critical that the dense wavelength division multiplexed transmission systems are being developed to meet the rapidly growing data traffic demands^58^. Our work can provide a novel ultrashort pulse sources to meet the requirements in the L band and also broaden the application wavelength of MoS_2_.

## Method

### Preparation of the MoS_2_-clad microfibre SA

The microfibre is manufactured using the fused biconical taper process. The fibre taper area has a minimum diameter of ~7 μm and is hung in the air with a glass holder. The monolayer MoS_2_ (from SixCarbon technol.) is grown on SiO_2_/Si substrate via chemical vapour deposition (CVD) method. Before transfer of MoS_2_, polymethylmethacrylate (PMMA) is spin-coated uniformly onto MoS_2_/SiO_2_/Si and dried for several hours. It comes into PMMA/MoS_2_/SiO_2_/Si, which is cut into strip samples with the width of ~0.3 mm and the length of ~4 mm. The samples are put into the KOH solution for 5 h to etch SiO_2_, so that MoS_2_ strips would separate from the substrate and float on the surface of the solution. The resulted PMMA/MoS_2_ strips are transferred into deionized water three times to rinse the etchant and residues. Then drop a small amount of alcohol to immerse the taper area where a sample is transferred with probes. The strips initially float on the alcohol and tightly cover onto the microfibre after the alcohol evaporates. The MoS_2_-clad microfibre is shown in Fig. 1(a).

### MCM-based fibre lasers and dispersion management

A schematic of the fibre laser mode-locked by MoS_2_ is shown in Fig. 2. The fibre laser system consists of a 50-m-long erbium-doped fibre (EDF) with 3 dB/m absorption around 1550 nm, which is pumped by a 980 nm laser diode (LD) via a polarization-independent tap-isolator-wavelength-division multiplexer (PI-TIWDM). Except of the WDM function, the compact integrated fibre device can be also used to extract intracavity power with a ratio of 10% and ensure the unidirectional operation. Pump power from a 980 nm laser diode is reflected and coupled into the common port. Laser centered around 1550 nm is inputted from signal port. 10% of input power is reflected and outputted from the tap port. 90% of input power passes through the inner isolator core and is coupled into the common port. Laser from common port cannot pass through the isolator core. The intracavity polarization controller (PC) is used to adjust the cavity linear birefringence to optimize the mode-locking performance. The MCM SA is used as the mode-locker to initiate the ultrashort pulse operation. The length of intracavity standard single-mode fibre (SMF) is changed to control the cavity dispersion. The EDF and SMF have dispersion parameters of approximately −25 and 17 ps/(nm·km) at 1550 nm, respectively.

The dispersion management is described as follows. Firstly, only EDF and fibre pigtails of component are used. At this time, the cavity length is ~56 m containing 50-m EDF and 6-m SMF and the total cavity dispersion is ~1.5 ps^2^. The total dispersion of 50-m EDF is ~1.6 ps^2^ which can be counterbalanced with ~70-m SMF. Here, ~70-m SMF is spliced into laser cavity. At this time, the cavity length is ~128 m and the total cavity dispersion is ~−0.1 ps^2^. If further lengthening the SMF, the laser cavity would display obvious anomalous dispersion. In the experiment, SMF is lengthened to ~100 m to provide enough anomalous dispersion. At this time, the cavity length is ~156 m and the total cavity dispersion is ~−0.7 ps^2^.

### Measurement method

An optical spectrum analyser (Yokogawa AQ-6370), an autocorrelator, a 6-GHz oscilloscope, a radio-frequency (RF) analyser, and a 3-GHz photodetector are used to measure the laser output performance.

## Additional Information

**How to cite this article**: Cui, Y. *et al*. MoS_2_-clad microfibre laser delivering conventional, dispersion-managed and dissipative solitons. *Sci. Rep.* **6**, 30524; doi: 10.1038/srep30524 (2016).
